# Supplementary material for: Structural absorption by barbule microstructures of super black bird of paradise feathers
Source: Nat Commun. 2018 Jan 9;9:1. doi: 10.1038/s41467-017-02088-w (PMC5760687; doi:10.1038/s41467-017-02088-w)
Supplement: Supplementary file 1 — Supplementary Information [file 41467_2017_2088_MOESM1_ESM.pdf]

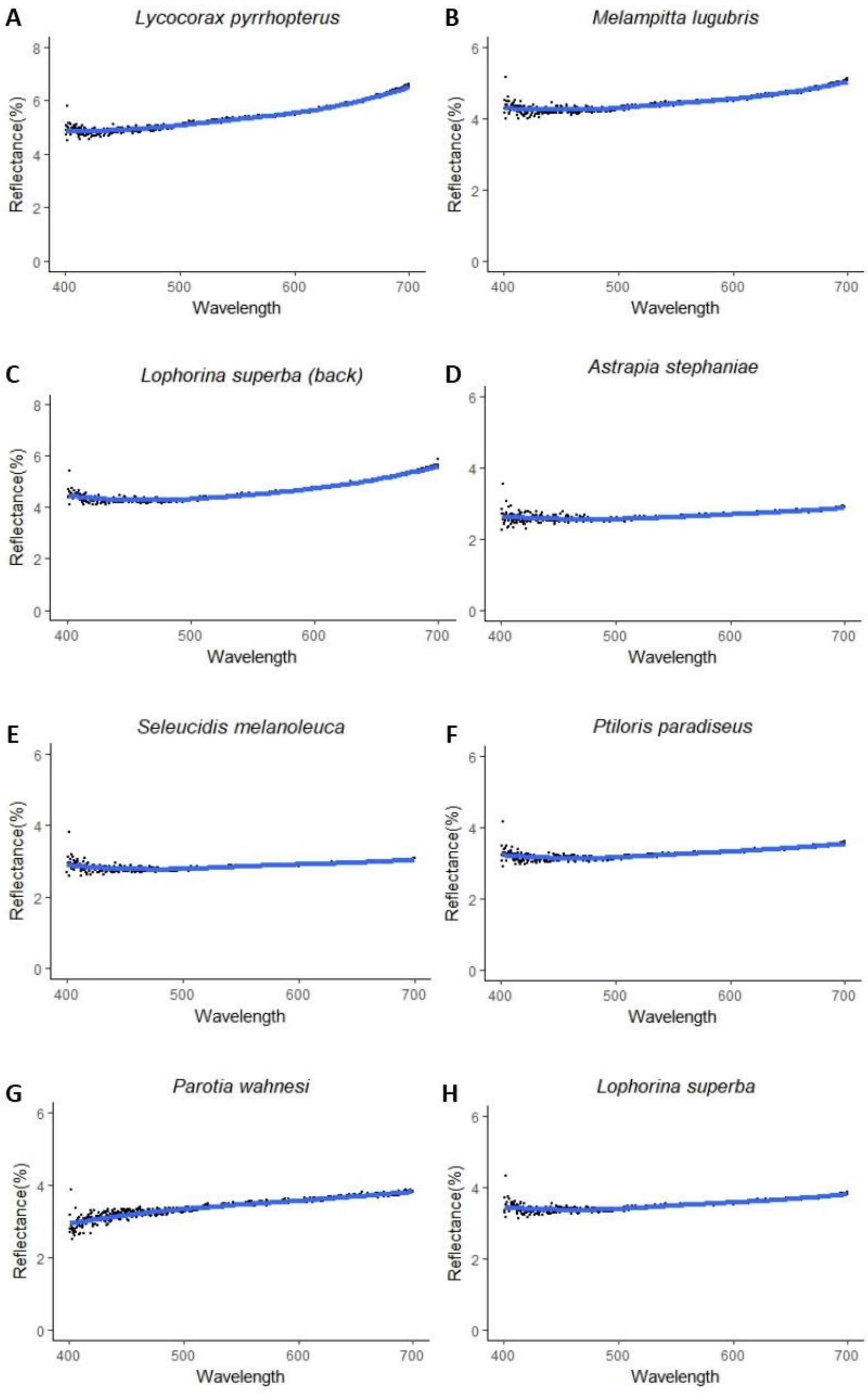

Supplementary Figure 1. Total reflectance spectra from integrating sphere of plumage patches. Spectra are averages of two specimens except for Astrapia and Parotia. Real data is plotted in black points, and Loess smoothing curve is shown in blue. (A) *Lycocorax pyrrhopterus* back. (B) *Melampitta lugubris* breast. (C) *Astrapia stephaniae* breast. (D) *Seleucidis melanoleuca* breast. (E) *Ptiloris paradiseus* breast. (F) *Parotia wahnesi* crown. (G) *Lophorina superba* cape.

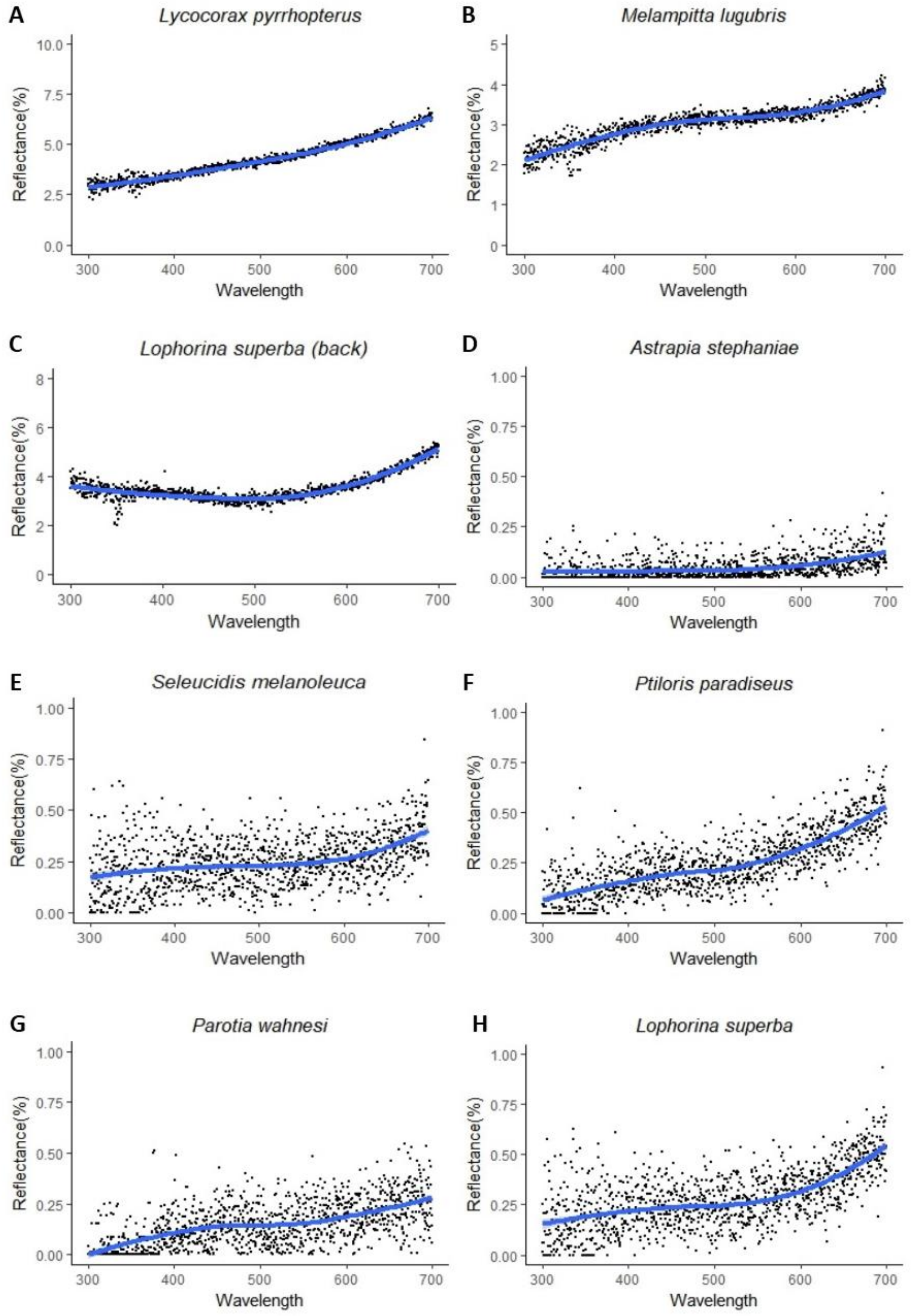

Supplementary Figure 2. Normal (0˚) directional reflectance. Each spectrum is the average of five measurements from one individual specimen. Real data is plotted in black points, and Loess smoothing curve is shown in blue. (A) *Lycocorax pyrrhopterus* back. (B) *Melampitta lugubris* breast. (C) *Lophorina superba* back. (D) *Astrapia stephaniae* breast. (E) *Seleucidis melanoleuca* breast. (F) *Ptiloris paradiseus* breast. (G) *Parotia wahnesi* crown. (H) *Lophorina superba* cape.

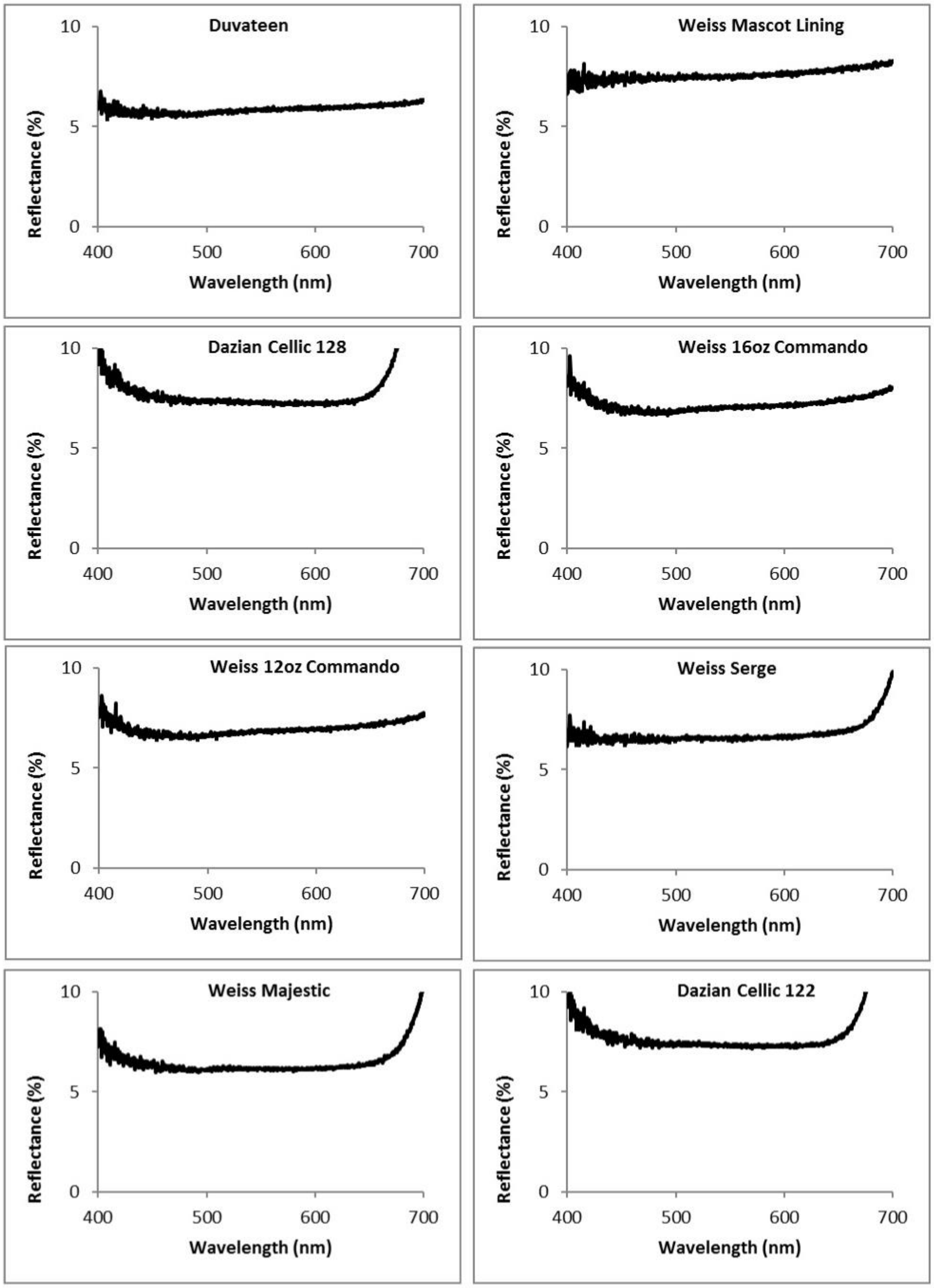

Supplementary Figure 3. **Total integrated diffuse and specular reflectance, using an integrating sphere, of commercially produced velvet.** The panels represent different brands and makes of velvet fabric, as labeled.

(A) *Lycocorax pyrrhopterus* back

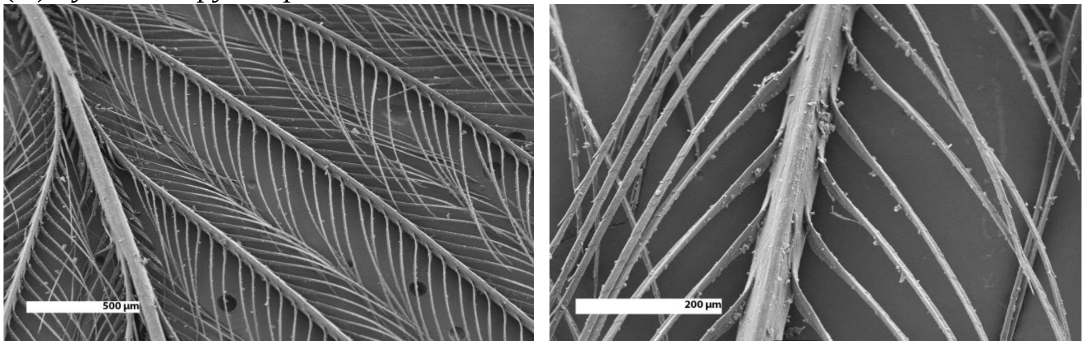

(B) *Melampitta lugubris* breast

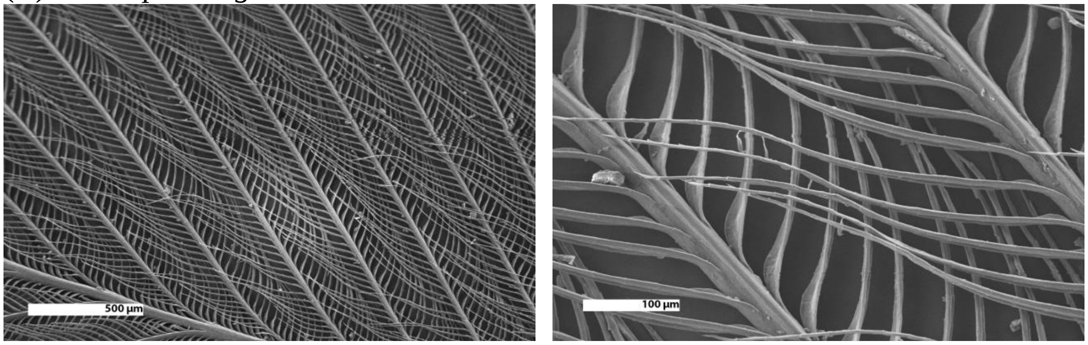

(C) *Astrapia stephaniae* breast

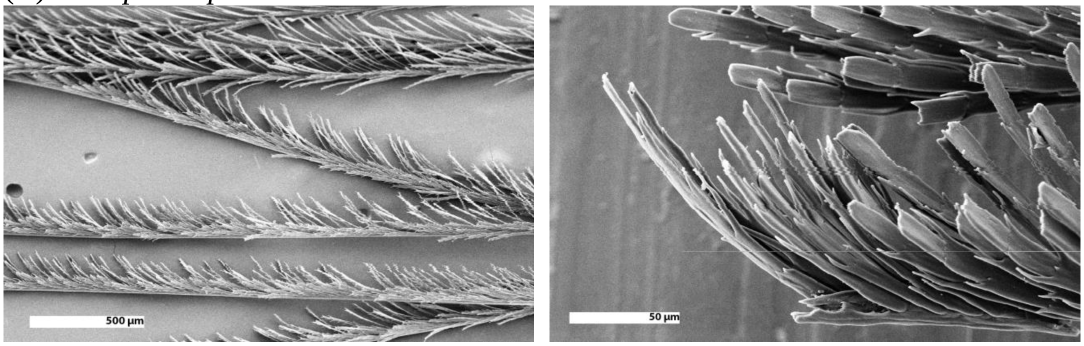

(D) *Seleucidis melanoleuca* breast

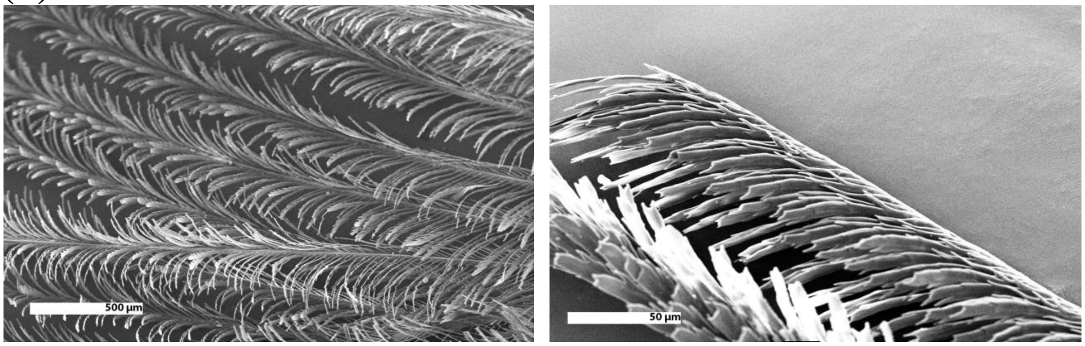

(E) *Ptiloris paradiseus* breast

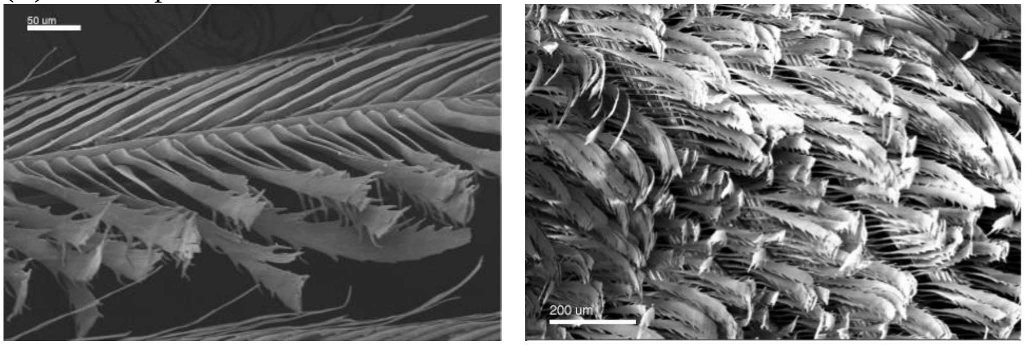

(F) *Parotia wahnesi* crown

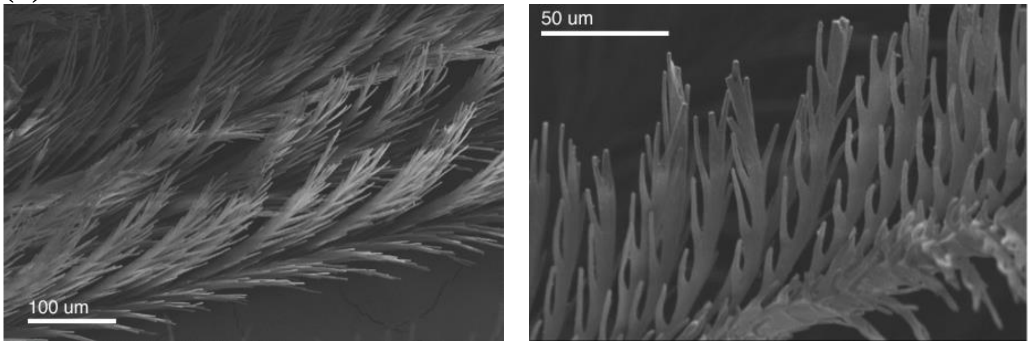

(G) *Lophorina superba* cape

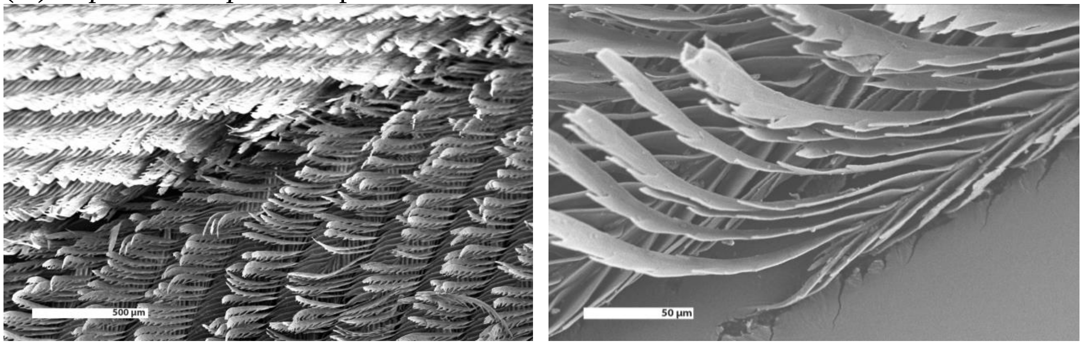

Supplementary Figure 4. SEM micrographs of the exposed tips of black feathers with normal barbules (A–B), and super black feathers with arrays of modified barbules (C–G). **(**A) *Lycocorax pyrrhopterus* back feather. (B) *Melampitta lugubris* breast feather. (C) *Astrapia stephaniae* breast feather. (D) *Seleucidis melanoleuca* breast feather. (E) *Ptiloris paradiseus* breast feather. (F) *Parotia wahnesi* crown feather. (G) *Lophorina superba* cape feather.

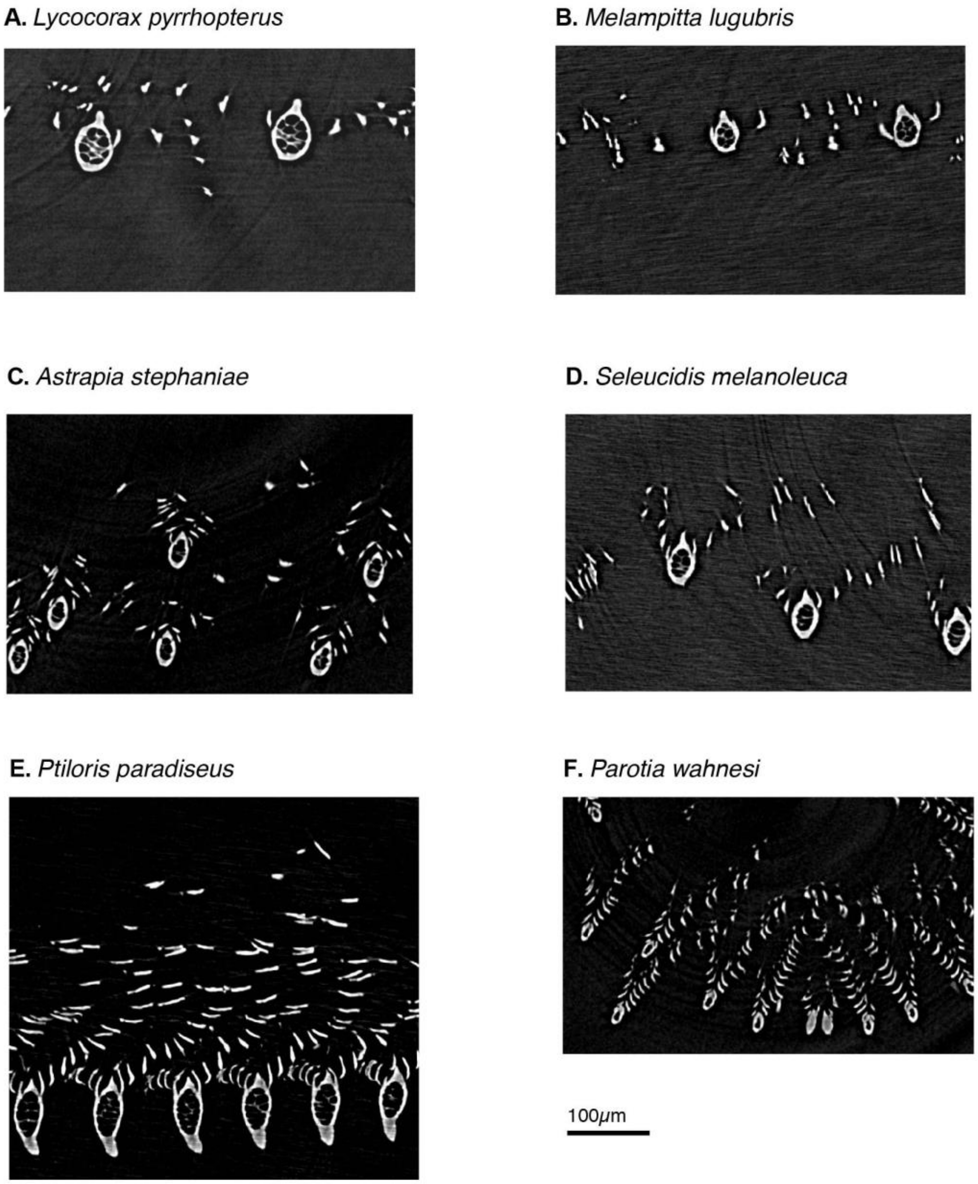

Supplementary Figure 5. Slices of high-resolution synchrotron X-ray data sets of the exposed tips of black feathers with normal barbules (A–B), and super black feathers with arrays of modified barbules (C–F). (A) *Lycocorax pyrrhopterus* back feather. (B) *Melampitta lugubris* breast feather. (C) *Astrapia stephaniae* breast feather. (D) *Seleucidis melanoleuca* breast feather. (E) *Ptiloris paradiseus* breast feather. (F) *Parotia wahnesi* crown feather. Scale bar is 100 µm.

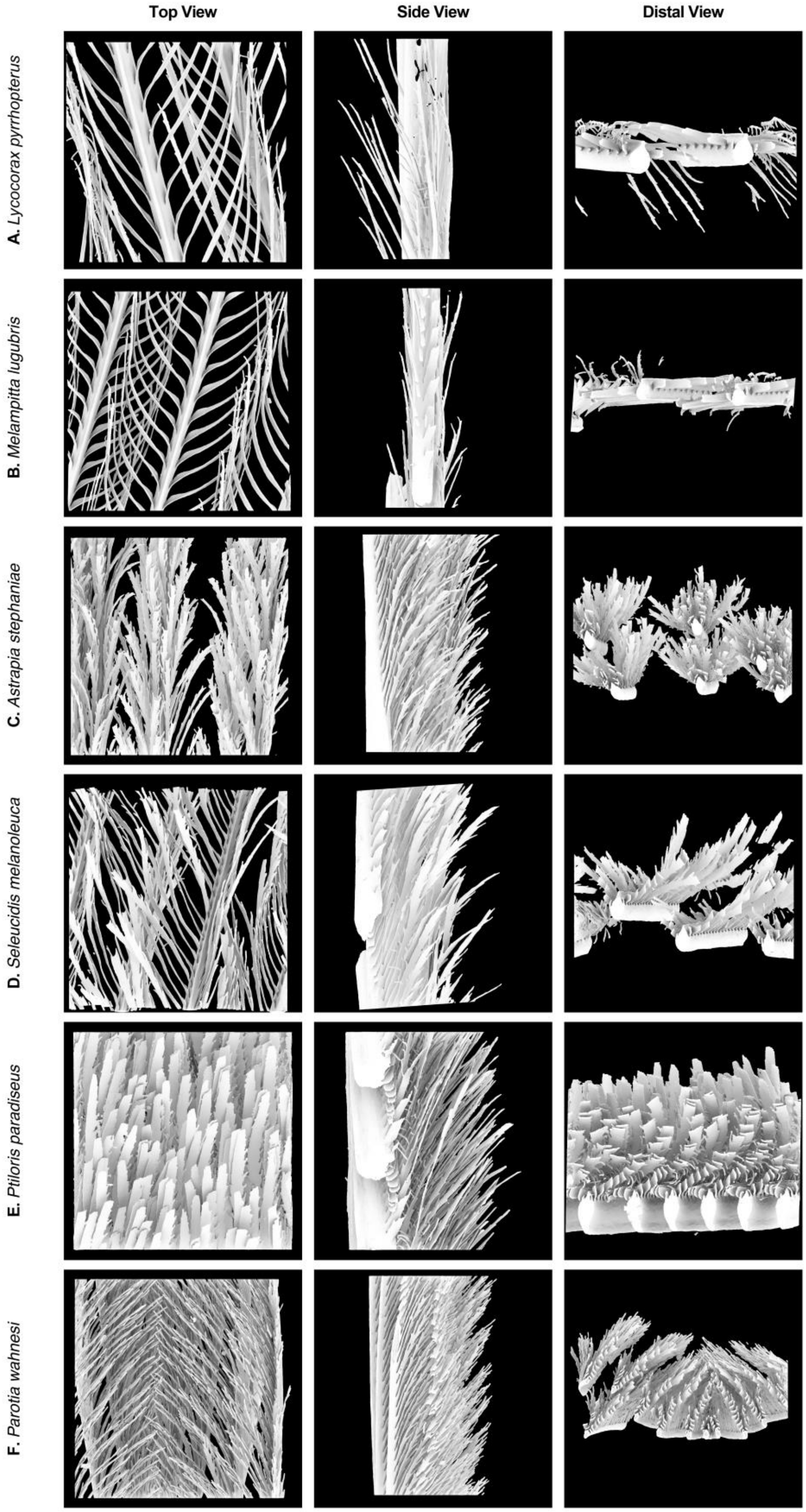
Top View
Side View
Distal View
A. Lycocorax pyrrhopterus
B. Melampitta lugubris
C. Astrapia stephaniae
D. Seleucidis melanoleuca
E. Ptiloris paradiseus
F. Parotia wahnesi

Supplementary Figure 6. 3D rendering of the external surface of feathers extracted from CT scans. View of the top or obverse feather surface (left), side view (middle), and distal view (right). Black feathers with normal barbules (A–B), and super black feathers with arrays of modified barbules (C–F). (A) *Lycocorax pyrrhopterus* back feather. (B) *Melampitta lugubris* breast feather. (C) *Astrapia stephaniae* breast feather. (D) *Seleucidis melanoleuca* breast feather. (E) *Ptiloris paradiseus* breast feather. (F) *Parotia wahnesi* crown feather. Each render is 500µm x 500µm

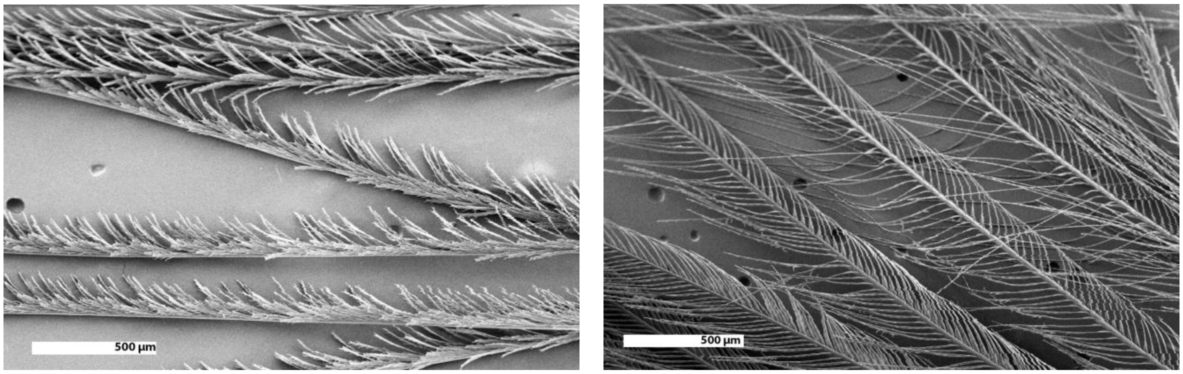

Supplementary Figure 7. Variation barbule morphology within the vane of a super black breast feather from *Astrapia stephaniae*. *Left*, Barbs and barbules from the *distal* tip of the feather with modified, structurally absorbing barbule arrays. *Right* Barbs and barbules from the proximal bases of the feather vane with normal barbule morphology. These barbules are deep within the plumage and are not modified for structural absorption. Scale bars are 500 µm.

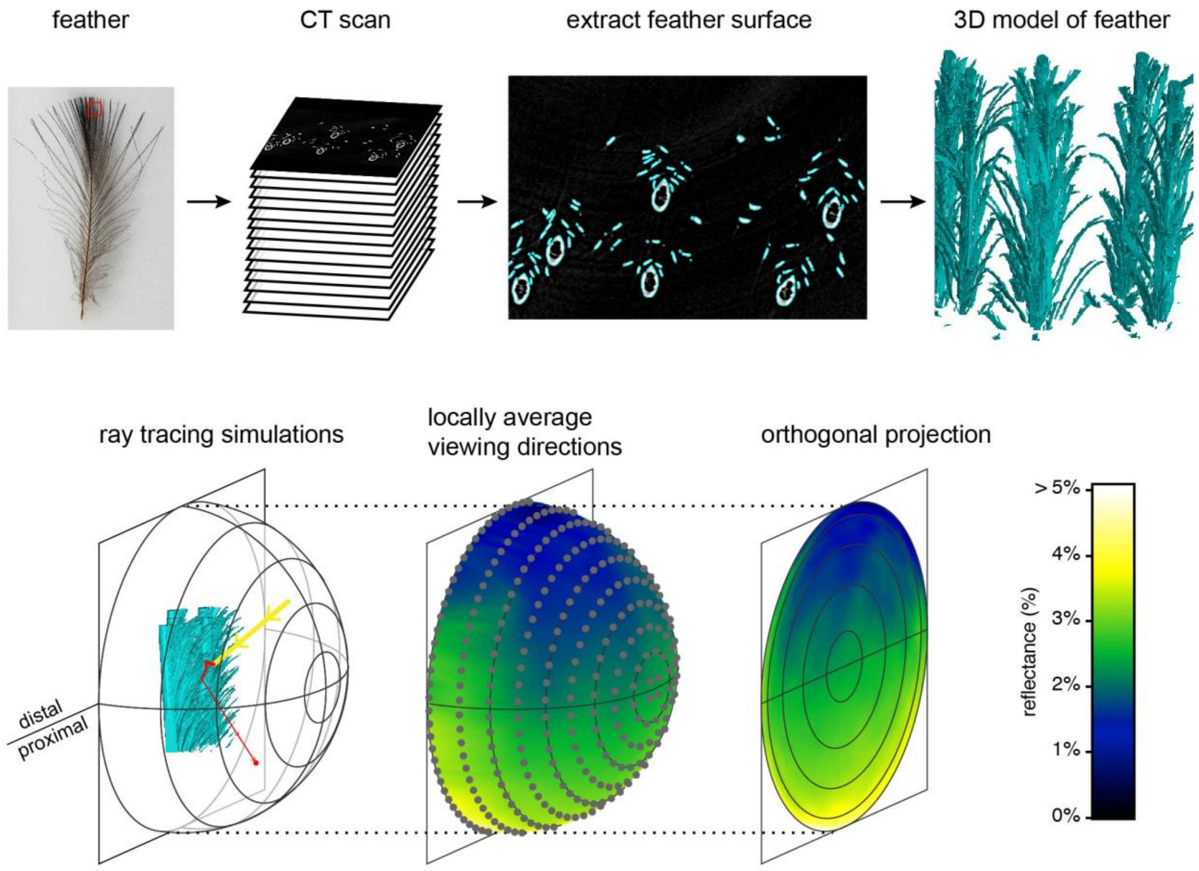

Supplementary Figure 8. Schematic of the ray tracing simulation methods. The distal tip of each feather was CT scanned at 650nm voxel resolution. The external surface of each feather was extracted from the CT image stack and converted into a 3D polygon mesh model. The 3D model was then imported into FRED for ray tracing simulations. The residual radiant power of rays that scattered off the surface of the feather and terminated on the reflectance hemisphere was tabulated. Locally averaged values for reflectance across the hemisphere were calculated for 400 evenly spaced viewing directions and plotted as a color gradient on orthogonal projections of the reflectance hemisphere.

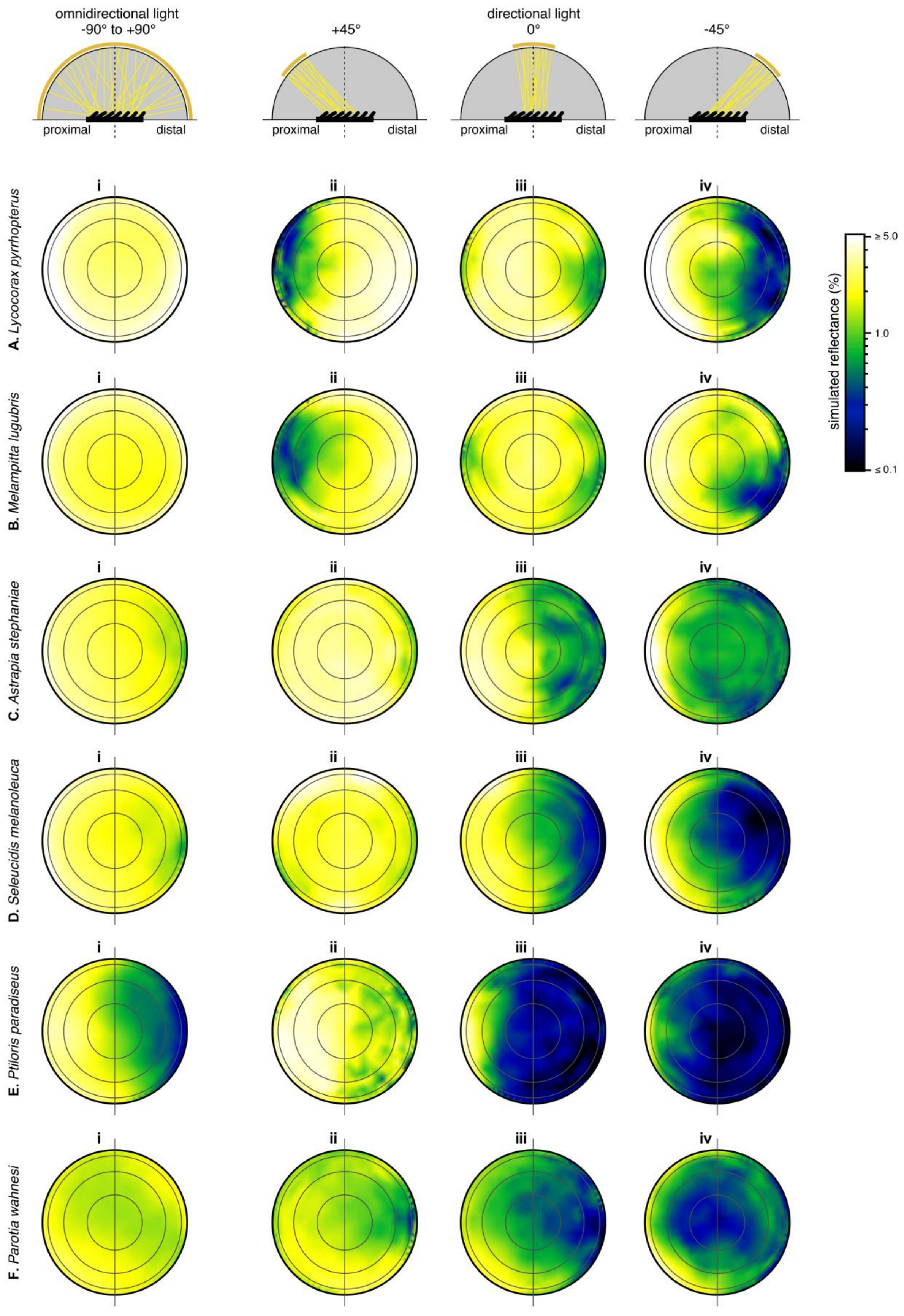
omnidirectional light
-90° to +90°
+45°
directional light
0°
-45°
proximal
distal
A. Lycocorax pyrrhopterus
B. Melampitta lugubris
C. Astrapia stephaniae
D. Seleucidis melanoleuca
E. Ptiloris paradiseus
F. Parotia wahnesi
i
ii
iii
iv
simulated reflectance (%)
≥ 5.0
1.0
≤ 0.1

Supplementary Figure 9. Directional reflectance of normal black and super black feathers under different lighting conditions, as predicted from ray tracing simulations. Schematics of each lighting setup along top; **i.** omnidirectional lighting with randomized incident light angles distributed across the entire reflectance hemisphere from –90° to +90°, **ii.** beam of incident light tilted +45° towards the proximal end of the feather, **iii.** beam of incident light oriented normal (0°) to the feather, and **iv.** beam of incident light tilted –45° towards the distal end of the feather. Directional reflectance is plotted as a log scale color gradient on orthogonal projections of the reflectance hemisphere. Concentric rings represent 22.5˚, 45˚, 67.5˚ and 90˚. (A) *Lycocorax pyrrhopterus* back feather. (B) *Melampitta lugubris* breast feather. (C) *Astrapia stephaniae* breast feather. (D) *Seleucidis melanoleuca* breast feather. (E) *Ptiloris paradiseus* breast feather. (F) *Parotia wahnesi* crown feather.

Supplementary Table 1: Specimen details, spectrophotometry, and ray tracing results

			Spectrophotometry		Ray Tracing Simulations		
**Species**	**Plumage Patch**	**Barbule Type**	**Total integrated Reflectance %**	**Normal Directional Reflectance %**	**Normal Directional Absorbance %**	**Normal Directional Multiple scattering %**	**Museum Catalog Number[1]**
Lycocorax pyrrhopterus	back	normal	5.39	4.68	96.72	33.66	YPM 75285, 75289, **75291**
Melampitta lugubris	breast	normal	4.47	3.23	97.02	40.42	YPM **75536**, 75537, 75538
Astrapia stephaniae	breast	modified array	3.14	0.05	97.81	55.39	YPM **7577**
Seleucidis melanoleuca	breast	modified array	3.18	0.26	98.98	80.62	YPM **75315,** 75317
Ptiloris paradiseus	breast	modified array	3.47	0.29	99.75	95.05	YPM **24130,** 94752
Parotia wahnesi	crown	modified array	3.43	0.18	99.46	89.53	AMNH 268233, **KU 93603**
Lophorina superba	cape	modified array	3.51	0.31	NA	NA	YPM 75359, 75360
Lophorina superba	back	normal	4.64	3.56	NA	NA	YPM 75359, 75360

[1] YPM – Yale Peabody Museum, MCZ – Harvard Museum of Comparative Zoology, KU – University of Kansas Biodiversity Institute, AMNH – American Museum of Natural History; catalog numbers in bold were used in ray tracing simulations.

Supplementary Table 2: Predicted directional reflectance (%) of distal and proximal quadrants from ray tracing simulations

	i. Omnidirectional		ii. Tilted +45° proximal		iii. 0° normal		iv. Tilted –45° distal	
	Proximal	Distal	Proximal	Distal	Proximal	Distal	Proximal	Distal
Lycocorax pyrrhopterus	4.33	3.73	2.33	4.71	3.45	2.25	4.36	1.03
Melampitta lugubris	3.36	2.89	1.66	3.41	2.51	1.92	3.28	1.19
Astrapia stephaniae	3.30	2.02	3.43	3.18	3.17	0.76	3.58	0.59
Seleucidis melanoleuca	3.40	1.95	3.18	2.76	2.60	0.53	2.80	0.28
Ptiloris paradiseus	2.74	0.74	4.00	1.60	2.08	0.19	0.90	0.19
Parotia wahnesi	1.91	1.64	1.62	1.40	1.33	0.58	1.36	0.56
